# BCI Suppresses RANKL-Mediated Osteoclastogenesis and Alleviates Ovariectomy-Induced Bone Loss

**DOI:** 10.3389/fphar.2021.772540

**Published:** 2021-11-01

**Authors:** Chenhui Cai, Wenhui Hu, Ying Zhang, Xu Hu, Sizhen Yang, Hao Qiu, Rujie Wang, Min Ma, Yiyun Qiu, Tongwei Chu

**Affiliations:** ^1^ Department of Orthopedics, Xinqiao Hospital, Third Military Medical University (Army Medical University), Chongqing, China; ^2^ Department of Biomedical Materials Science, Third Military Medical University (Army Medical University), Chongqing, China

**Keywords:** osteoclast, bone resorption, postmenopausal osteoporosis, bioinformatics, BCI

## Abstract

Osteoporosis is a common aging-related metabolic disease that mainly occurs in older adults and postmenopausal women. Despite advances in anti-osteoporosis treatment, outcomes remain unsatisfactory due to detrimental side effects. BCI hydrochloride (BCI), a selective dual-specificity phosphatase 6 (DUSP6) inhibitor, is associated with multiple cellular functions, including inhibiting tumor growth and macrophage inflammation; however, its role in regulating osteoclast differentiation remains unknown. Here, we revealed that treatment with BCI attenuated RANKL-mediated osteoclast differentiation *in vitro* and alleviated ovariectomy-induced osteoporosis without obvious toxicity. Specifically, BCI disrupted F-actin ring formation and bone-resorption activity and decreased osteoclast-specific gene and protein levels in a dose-dependent manner. KEGG pathway analysis, GSEA based on transcriptome sequencing, and western blot results suggested that BCI inhibited RANKL-induced osteoclastogenesis by restraining STAT3 and NF-κB signaling and attenuating NF-κB/p65 interaction with NFATc1. These results revealed that BCI treatment prevented postmenopausal osteoporosis and might represent an effective approach for treating osteoporosis.

## Introduction

Osteoporosis is a common and frequent skeletal disorder due to an imbalance in bone remodeling associated with excessive osteoclast activity and inadequate osteoblast generation (Henriksen et al., 2011; Rachner et al., 2011). Osteoclast precursors are originated from the monocyte/macrophage lineage of hematopoietic stem cells and gradually differentiate into mature osteoclast in the presence of macrophage colony-stimulating factor (M-CSF) and receptor activator of nuclear factor-kappaB (NF-κB) ligand (RANKL) (Kearns et al., 2008). During osteoclastogenesis, M-CSF binding to CSF-1 receptor is responsible for ensuring cell proliferation, whereas RANKL interaction with its receptor RANK promotes osteoclast maturation and resorption (Sobacchi et al., 2013). RANKL–RANK interaction on the surface of preosteoclasts leads to activation of a range of downstream signals, including those related to NF-κB, mitogen-activated protein kinase (MAPK), and signal transducer and activator of transcription 3 (STAT3), that initiate osteoclast activation, differentiation, and function (Kim et al., 2017). Similarly, STAT3 acts a significant role during osteoclastogenesis by regulating the NF-κB pathway (Shankar et al., 2012). These pathways eventually work together to activate critical transcription factors in osteoclastogenesis, such as c-Fos and NFATc1 (Nakashima et al., 2012). Therefore, inhibition of these pathways might contribute to osteoporosis treatment. Recently, new agents targeting osteoclast bone-resorption activity or osteoblastic bone formation, including bisphosphonates, calcitonin, denosumab, selective estrogen receptor modulators, and teriparatide, have been widely developed, explored, and utilized to prevent and treat osteoporosis (Khalili et al., 2012; Leder et al., 2015). However, most of these therapeutic approaches lead to serious side effects, which discourage their long-term use and patient compliance (Li et al., 2019). Thus, there is an urgent need to develop alternative approaches to treating osteoporosis with improved effectiveness and lower toxicity.

Dual-specificity phosphatases (DUSPs) are protein phosphatases responsible for dephosphorylating molecules at tyrosine and serine/threonine residues in a single substrate (Ahmad et al., 2018). There are >20 known DUSPs in mammals, of which DUSP6 functions as either an oncogene or tumor-suppressor according to different tumor types. Additionally, DUSP6 is closely associated with critical biological processes, such as cardiac development, knot proctitis, and embryonic development (Li et al., 2007; Maillet et al., 2008; Bertin et al., 2015). Several studies report that DUSP6 also acts as a mediator of inflammation-associated T cell activation and differentiation (Li et al., 2015; Hsu WC. et al., 2018). BCI hydrochloride (BCI) is a small-molecule inhibitor that reportedly attenuates DUSP6 levels and promotes the expression of fibroblast growth factor (FGF) in zebrafish embryos (Molina et al., 2009). Additionally, an *in vitro* study found that BCI attenuates lipopolysaccharide (LPS)-induced proinflammatory responses in macrophages by activating Nrf2 (Zhang et al., 2019). Due to its close association with inflammatory signals, we hypothesized that BCI might affect osteoclast differentiation.

Our research for the first time investigated the ability of BCI to restrain RANKL-induced osteoclastogenesis, as well as the associated regulatory mechanisms. We demonstrated that BCI suppressed the formation, fusion, and bone-resorbing activity of osteoclasts in a concentration-dependent way. Furthermore, we found that BCI-mediated DUSP6 inhibition attenuated bone loss in an ovariectomy (OVX)-induced osteoporosis mouse model. These findings suggest that BCI treatment might represent a new approach for treating osteoporosis.

## Materials and Methods

### Drugs and Reagents

BCI hydrochloride (also called BCI or (E)-BCI)) was obtained from MCE (Shanghai, China) and dissolved in DMSO. DMEM, α-MEM, and FBS were purchased from Hyclone (UT, United States). Recombinant murine RANKL and M-CSF were purchased from R&D (MN, United States). Primary antibodies specific for β-tubulin, glyceraldehyde 3-phosphate dehydrogenase (GAPDH), phospho-NF-kB/p65, NF-kB/p65, p-IkBα, IkBα, p38, p-ERK, ERK, CTSK, MMP9, and c-Fos were obtained from Proteintech (Wuhan, China). Primary antibodies specific for p-JNK, JNK, and p-p38 were obtained from Affinity Biosciences (Zhenjiang, China). Primary antibodies specific for p-STAT3, and STAT3 were from Bioworld (Nanjing, China). Antibodies against NFATc1 were obtained from Santa Cruz (TX, United States). Anti-rabbit and anti-mouse HRP-labeled secondary antibodies were obtained from Beyotime (Shanghai, China).

### Cell Culture and Osteoclast Differentiation

The murine macrophage cell line RAW264.7 was derived from ATCC Cell Bank (Shanghai, Chinese) and grown in DMEM (10% FBS and 1% P/S). Bone marrow monocytes/macrophages (BMMs) were isolated from the femurs or tibias of mice aged 4–6 weeks and maintained in α-MEM (10% FBS, 1% P/S, and 50 ng/ml M-CSF) for 48 h. All cells were grown at 37°C under 5% CO_2_. Both cell types were maintained in M-CSF (50 ng/ml) and RANKL (100 ng/ml) for osteoclast differentiation. Osteoclastogenesis of RAW264.7 cells was measured at 3 days and that of BMMs at 5 days. MC3T3-E1 cells (a preosteoblast cell line) were maintained in α-MEM (10% FBS and 1% P/S) and cultured in osteogenesis-inducing fluid, which included ascorbic acid (60 μg/ml) and β-glycerophosphate (6 mM) for osteoblastogenesis.

### Cytotoxicity Assays

Cell Counting Kit-8 (CCK-8) test was carried out to determine the appropriate concentrations of BCI for evaluating cell toxicity. RAW264.7 cells and BMMs were grown in 96-well plates, and BCI toxicity was assessed using doses ranging from 0 to 8 μM for 1 or 3 days. The cells were then washed in PBS and cultured in CCK-8 solution for 1.5–2 h. Absorbance was read at 450 nm using a spectrophotometer (Bio-Tek, VT, USA).

Flow cytometry was carried out to evaluate the apoptosis and cell cycle progression of 1.5 × 10^6^ BMMs grown in differentiation medium with or without BCI (2–4 μM) for 3 days. Cells were washed with PBS and fixed in 70% ethanol, followed by propidium iodide (PI) staining as a standard procedure and detection using the FACStar flow cytometer (Becton Dickinson, NJ, USA). For apoptosis rate evaluation, cells were stained with Annexin V/PI and Annexin V/fluorescein isothiocyanate, followed by detection using the FACStar flow cytometer (Becton Dickenson).

### TRAP Staining

RAW264.7 cells and BMMs were grown in 96-wells plates and induced to osteoclasts, as previously described. Upon observation of multi-nucleated osteoclasts, TRAP staining was performed according to manufacturer protocol (TRAP staining kit (Sigma, MO, USA). Cells were counted using an optical microscope (Leica Microsystems, Wetzlar, Germany), and TRAP + cells including three or more nuclei were considered mature osteoclasts.

### Focal Adhesion Kinase Staining Assay

Cells in 96-well plates were rinsed with PBS and fixed for 30 min using 4% PFA at 37°C, followed by washing twice with wash buffer and permeabilization with Triton X-100. Cells were then washed again and blocked with 2% BSA for 20 min. After being stained with tetramethylrhodamine-conjugated phalloidin for 1.5 h, the cells were washed twice with wash buffer. Nuclear counterstaining was performed with DAPI. The fluorescence images were visualized by fluorescence microscopy (Nikon, Tokyo, Japan).

### Pit Formation Assay

BMMs were generated on bovine cortical bone slices and induced according to experimental requirements. After rinsing with sodium hypochlorite, the bone slices were incubated in toluidine blue for 5 min. Images were collected using an inverted microscope (Leica Microsystems), and the relative resorption area was determined by ImageJ.

### Alkaline Phosphatase and Alizarin Red S Staining

MC3T3-E1 cells were cultured on 24-well plates and maintained in an osteogenic medium. Cells were fixed for 30 min in 70% ethanol and rinsed twice with deionized water. ALP activity was assessed using the NBT/BCIP substrate system (Sigma-Aldrich). For alizarin red S staining, cells were prepared, as previously described, and then stained in alizarin red stain solution. Images were acquired using an inverted microscope.

### RNA Extraction and qRT-PCR

RNA was obtained using TRIzol buffer, and concentrations were determined using a NanoDrop ND-1000 microplate reader (Thermo, Loughborough, UK). A260/A280 measurements were used to assess RNA purity, and cDNA was synthesized using PrimeScript^TM^ Reverse Transcriptase (Takara, Japan). For qRT-PCR, 2 µl of cDNA was mixed with SYBR Green super mix and a primer pair (Sangon Biotech). The expression of DUSP6, CTSK, c-Fos, MMP9, NFATc1, CD9, OSCAR, PU.1, ATP6V0d2, RUNX2, COL1α1, and ALPL was assessed, with GAPDH used as a reference. All primer sequences are listed in Supplementary Table S1.

### RNA Sequencing Transcriptomics and Bioinformatics Analyses

Gene expression in RAW264.7 cells induced in differentiation medium with or without BCI (2 μM) for 3 days was determined by RNA-seq analysis. Assays were performed in triplicate, and sequencing was performed by Sangon Biotech using a HiseqTM 2500 system (Illumina). Data analyses were carried out using the DEGseq R package (Wang et al., 2010), and differentially expressed genes (DEGs) were identified with thresholds of |logFC| >1 and an adjusted *p* < 0.05. Gene Ontology (GO) annotations and Kyoto Encyclopedia of Genes and Genomes (KEGG) pathway analysis of the DEGs were carried out using the clusterProfiler R package (Yu et al., 2012). Gene Set Enrichment Analysis (GSEA) (Powers et al., 2018) was performed using the h. all.v7.2. symbols.gmt reference gene set, with *p* < 0.05 and false discovery rate (FDR) < 0.25 considered statistically significant.

### Western Blot

Cells were washed in pre-cooled PBS and lysed on ice with RIPA buffer plus the protease inhibitor and phosphatase inhibitors for 30 min. At least 40 µg of proteins were electrophoresed by SDS-PAGE gels and then transferred to PVDF membrane. Blocking was performed with 4% BSA in TBST for 2 h, followed by probing of the membranes with specific primary antibodies (1:1,000) overnight and washing with TBST four times. After incubation with HRP-linked secondary antibodies for 1.5 h and washing with TBST four times, images were obtained using a Bio-Rad imaging system (CA, United States) and ECL luminous fluid.

### Nuclear Translocation of p65 and NFATc1

To observe the impact of BCI on the nuclear translocation of NFATc1 and NF-kB/p65 during RANKL-induced osteoclastogenesis, RAW264.7 cells pretreated with or without 2 μM BCI for 2 h were induced with 100 ng/ml RANKL for 20 min, followed by fixation in 4% PFA, permeabilization with Triton X-100, and blocked with 4% BSA for 1.5 h. The cells were then probed in specific primary antibodies overnight at 4°C, followed by incubation with Alexa Fluor 647-labeled secondary antibodies (Proteintech). Nuclei counterstaining was carried out with DAPI. Fluorescence images were visualized by confocal microscopy (Leica Microsystems), and fluorescence intensities were analyzed with ImageJ.

### Co-IP Assays

RAW264.7 was induced in a differentiation medium with or without BCI (1–2 μM) for 3 days, followed by incubation with lysis buffer on ice for 1 h and centrifugation at 12,000*g*. Immune complexes were formed by adding anti-NF-kB/p65 (1:150) to the cell lysate and incubating overnight at 4°C. IP of NF-kB/p65 was carried out using protein A and G magnetic beads (Thermo, MA, USA) per manufacturer instructions. IP complexes were obtained by incubating with the magnetic beads for 60 min under room temperature. Then magnetic beads were then collected on a magnetic stand, washed three times in IP buffer, and the immunocomplexes were washed with elution buffer and detected using western blot.

### Establishment of the OVX Mouse Model of Osteoporosis

Female C57BL/6 mice (8-weeks old) were acquired from the Animal Center of the Army Medical University (Chongqing, China) and randomized into groups: Sham, OVX, OVX + low-dose (15 mg/kg) BCI, and OVX + high-dose (30 mg/kg) BCI groups. After anesthetization by intraperitoneal injection of 5% chloral hydrate, the mice were bilaterally ovariectomized to establish the osteoporosis model. Mice in the Sham group underwent a sham operation. After recovering, mice from different groups were administered BCI (15 or 30 mg/kg) or saline by intraperitoneal injection for 7 weeks. The mice were housed in an SPF environment in the Animal Center of the Army Medical University. Experiments were approved by the Ethics Committee of the Army Medical University and carried out based on its guidelines.

### Micro-Computed Tomography and Histologic Assessment

After euthanasia, femurs of mice were fixed with 4% PFA for micro-CT scanning (Bruker, Kontich, Belgium), after which all of the samples were decalcified using a 10% EDTA solution for 10 days. H&E, Masson, and TRAP staining were then carried out as previously described. Sections were observed using an inverted microscope (Leica Microsystems), and morphological evaluation of liver and kidney tissues was performed via H&E staining.

### Statistical Analysis

Data are presented as the mean ± standard deviation, and statistical analysis was carried out using GraphPad Prism. Student’s *t*-tests were used to comparison of two groups, and one-way ANOVA was used to comparison of three or more groups. A *p* < 0.05 was considered significant.

## Results

### The Effects of BCI Treatment on Cell Cycle, Viability, and Apoptosis

**FIGURE 1:**
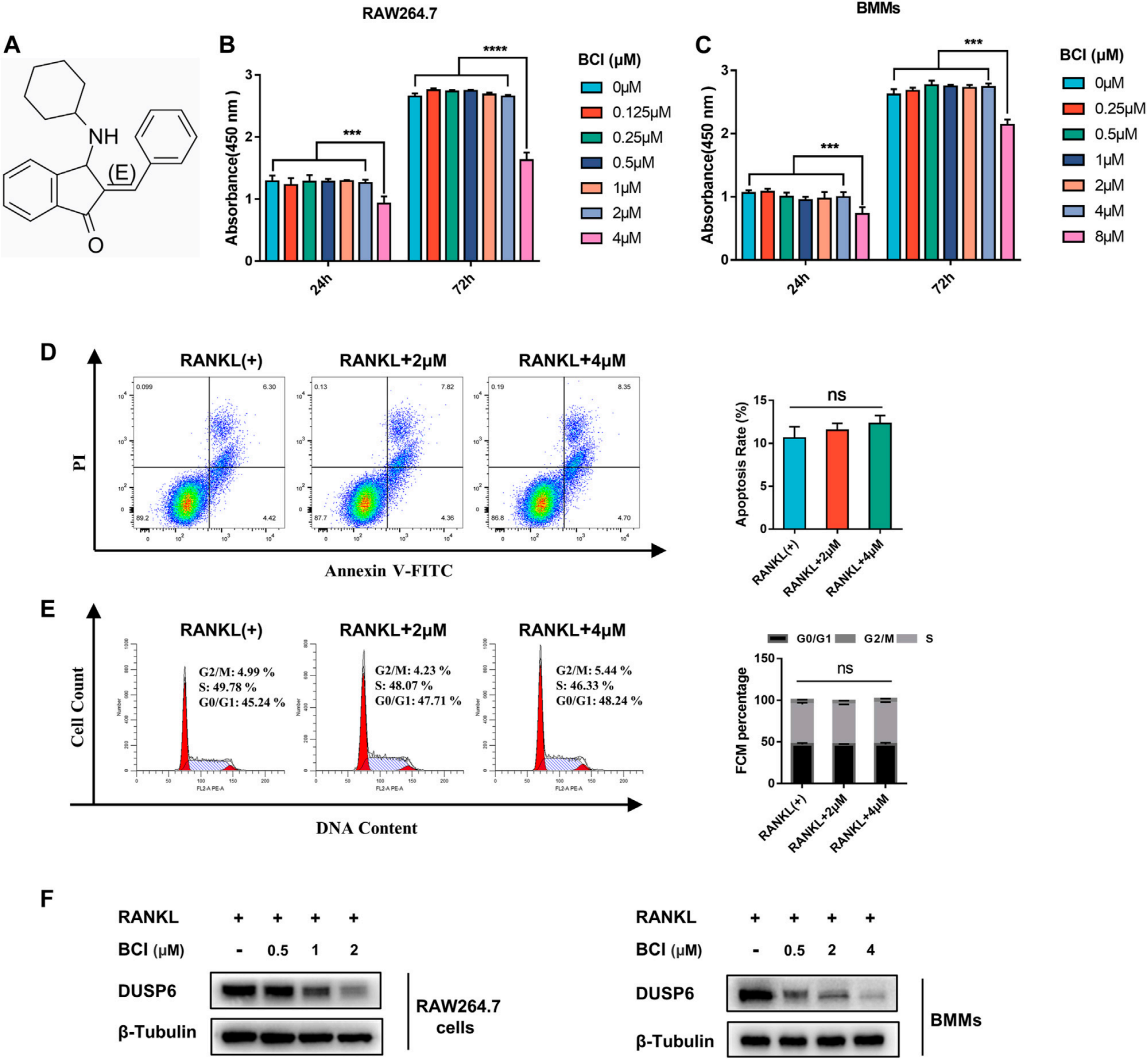
The effects of BCI treatment on cell cycle, viability, and apoptosis. **(A)** Chemical structure of BCI. **(B,C)** RAW264.7 cells and BMMs induced by RANKL and M-CSF were treated with various concentrations of BCI for 1 and 3 days, followed by CCK-8 assays to assess cell viability. **(D,E)** Flow cytometric analysis to evaluate apoptosis and cell cycle progression of BMMs treated with or without BCI. **(F)** Confirmation of BCI-mediated suppression of DUSP6 expression in RAW264.7 cells and BMMs during RANKL-mediated osteoclast differentiation. *p < 0.05, **p < 0.01, ***p < 0.001.

The chemical structure of BCI is displayed in Figure 1A. First, we assessed BCI toxicity to RAW264.7 cells and BMMs during treatment for 1 and 3 days, with the results revealing that low-dose BCI (≤2 μM and ≤4 μM) showed no cytotoxic effects on RAW264.7 cells and BMMs, respectively (Figures 1B,C). To further evaluate the effect of BCI on apoptosis and cell cycle progression, we carried out flow cytometric. The results indicated that BCI (≤4 μM) had no obvious effect on cell cycle progression (Figure 1D) or apoptosis (Figure 1E) in BMMs. We subsequently confirmed that BCI suppressed DUSP6 protein expression in RAW264.7 cells and BMMs during RANKL-mediated osteoclast differentiation (Figure 1F). These results indicated that BCI inhibited DUSP6 expression but does not affect cell viability, cell cycle progression, or apoptosis at certain concentrations.

### BCI Suppresses RANKL-Mediated Osteoclast Formation

**FIGURE 2:**
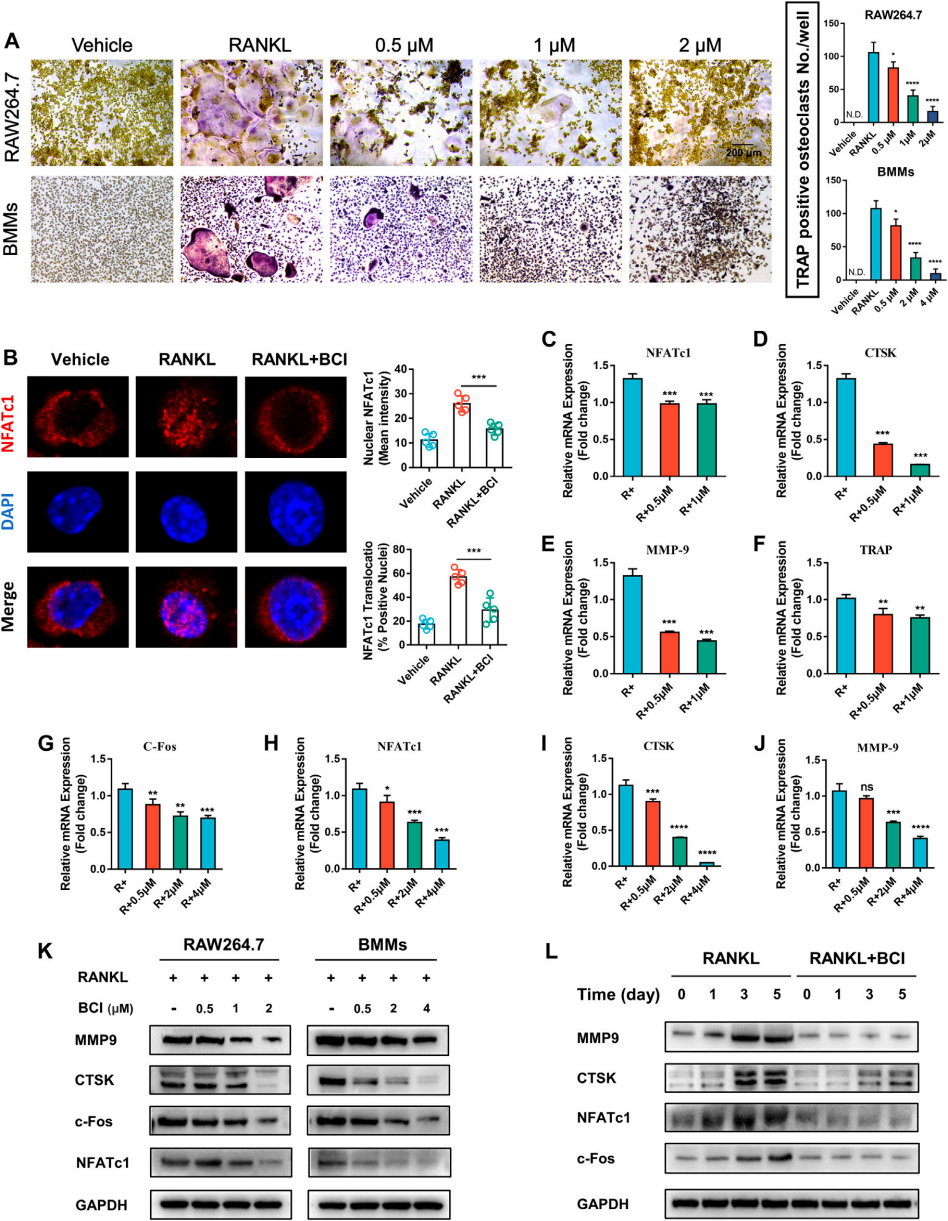
BCI suppresses RANKL-induced osteoclast differentiation. **(A)** TRAP staining results showing the effects of different doses of BCI on osteoclast differentiation. **(B)** Immunofluorescence staining of NFATc1 nuclear translocation according to BCI treatment. qRT-PCR analysis of the expression of osteoclast-specific genes (CTSK, MMP9, NFATc1, and c-Fos) during osteoclastogenesis in BCI-treated **(C–F)** RAW264.7 cells and **(G–J)** BMMs. **(K)** Western blot analysis of osteoclast marker levels during RANKL-induced osteoclastogenesis and at different BCI dosages. **(L)** Western blot analysis of osteoclast marker levels during RANKL-induced osteoclastogenesis following BCI treatment for the indicated times. *p < 0.05, **p < 0.01, ***p < 0.001.

To determine whether BCI-mediated DUSP6 inhibition suppresses RANKL-mediated osteoclast differentiation, we performed TRAP staining of RAW264.7 cells and BMMs. Figure 2A shows that BCI treatment attenuated osteoclast formation in a concentration-dependent way. Previous studies report that during osteoclast activation, NFATc1 translocates from cytoplasm to nucleus and transcribes downstream osteoclast-specific genes (Henriksen et al., 2011). To evaluate the effect of DUSP6 inhibition on NFATc1 nuclear translocation, RAW264.7 cells pretreated with or without 2 μM BCI for 2 h were induced with 100 ng/ml RANKL for 20 min. Our results indicated that RANKL-mediated NFATc1 nuclear translocation was repressed by BCI treatment (Figure 2B). Moreover, qRT-PCR evaluation of BCI-specific effects on osteoclast-specific genes expression (CTSK, MMP9, NFATc1, and c-Fos) revealed significant downregulation of their mRNA levels following BCI treatment of RAW264.7 cells (Figures 2C–F) and BMMs (Figures 2G–J) in a concentration-dependent manner. Western blot subsequently confirmed a similar dose-dependent downregulation of osteoclastic maker proteins following BCI exposure (Figures 2K,L).

### BCI Attenuates RANKL-Mediated Osteoclast Fusion and Resorption

**FIGURE 3:**
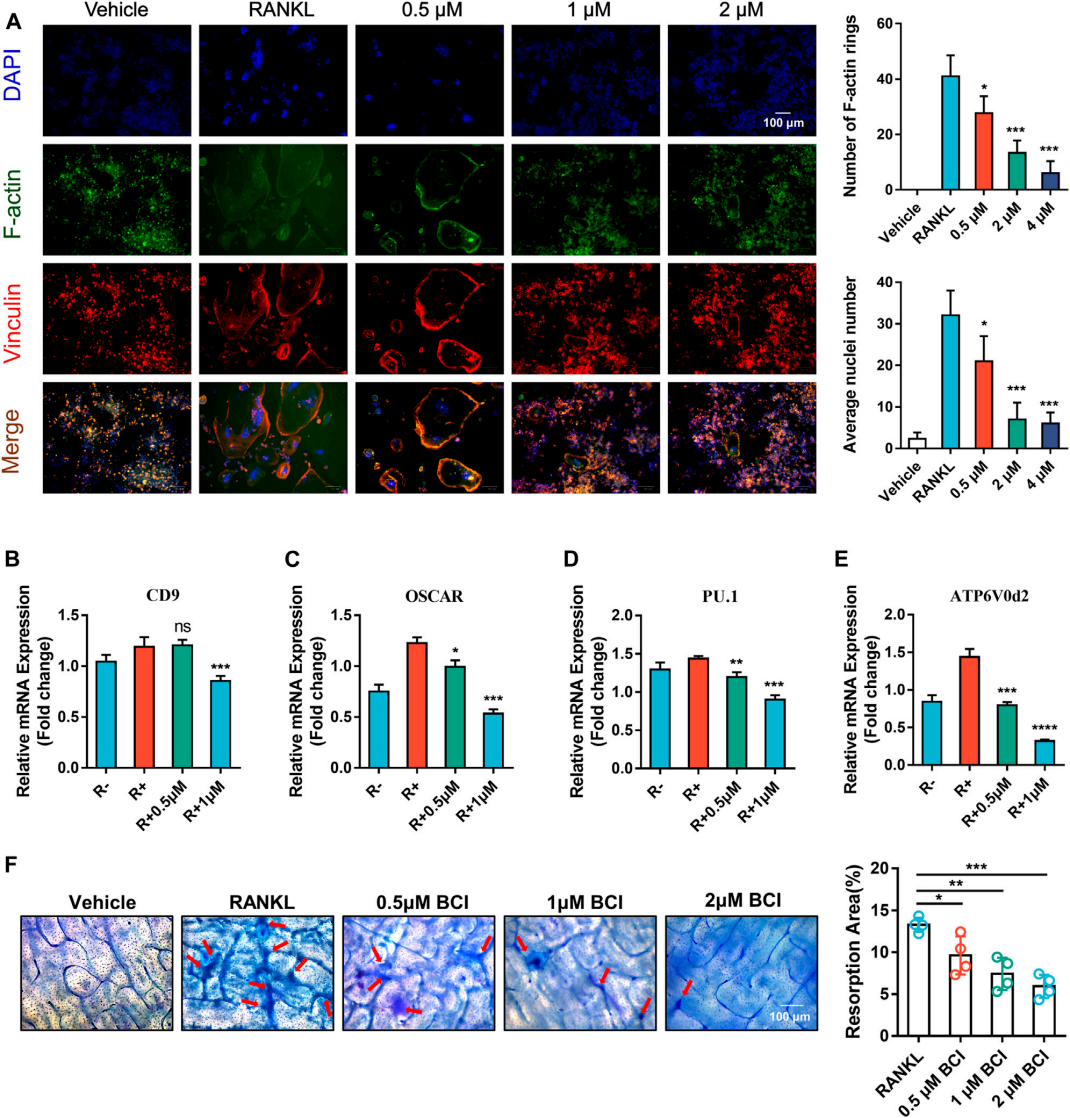
BCI inhibits RANKL-mediated osteoclast fusion and resorption. **(A)** Images of FAK staining of RANKL-induced RAW264.7 cells and treated with or without different dosages of BCI. Scale bars, 100 µm. **(B–E)** qRT-PCR analysis of the expression of osteoclast-fusion genes (ATP6v0d2, PU.1, OSCAR, and CD9) during RANKL-induced osteoclastogenesis in the presence of BCI. **(F)** Pit-formation assay assessing the effect of BCI on RANKL-induced osteoclast bone-resorption activity. Scale bars, 100 µm. The significant difference between the RANKL group and BCI-treated groups was expressed as*p < 0.05, **p < 0.01, ***p < 0.001.

To further evaluate the impact of DUSP6 inhibition on osteoclastic fusion, we carried out FAK staining of induced RAW264.7 treated with various doses of BCI. FAK staining indicated obvious reductions in the number of F-actin rings following BCI treatment (Figure 3A). Additionally, qRT-PCR results demonstrated that mRNA levels of osteoclast-fusion genes (ATP6v0d2, PU.1, OSCAR, and CD9) were reduced by BCI treatment (Figures 3B–E), which agreed with FAK-staining results. For the pit-formation assay, BMMs were induced in differentiation medium with or without BCI for 7 days, with quantitative results revealing marked reductions in the number of resorption pits under BCI treatment (Figure 3F).

### Transcriptome Analysis Following BCI Treatment

**FIGURE 4:**
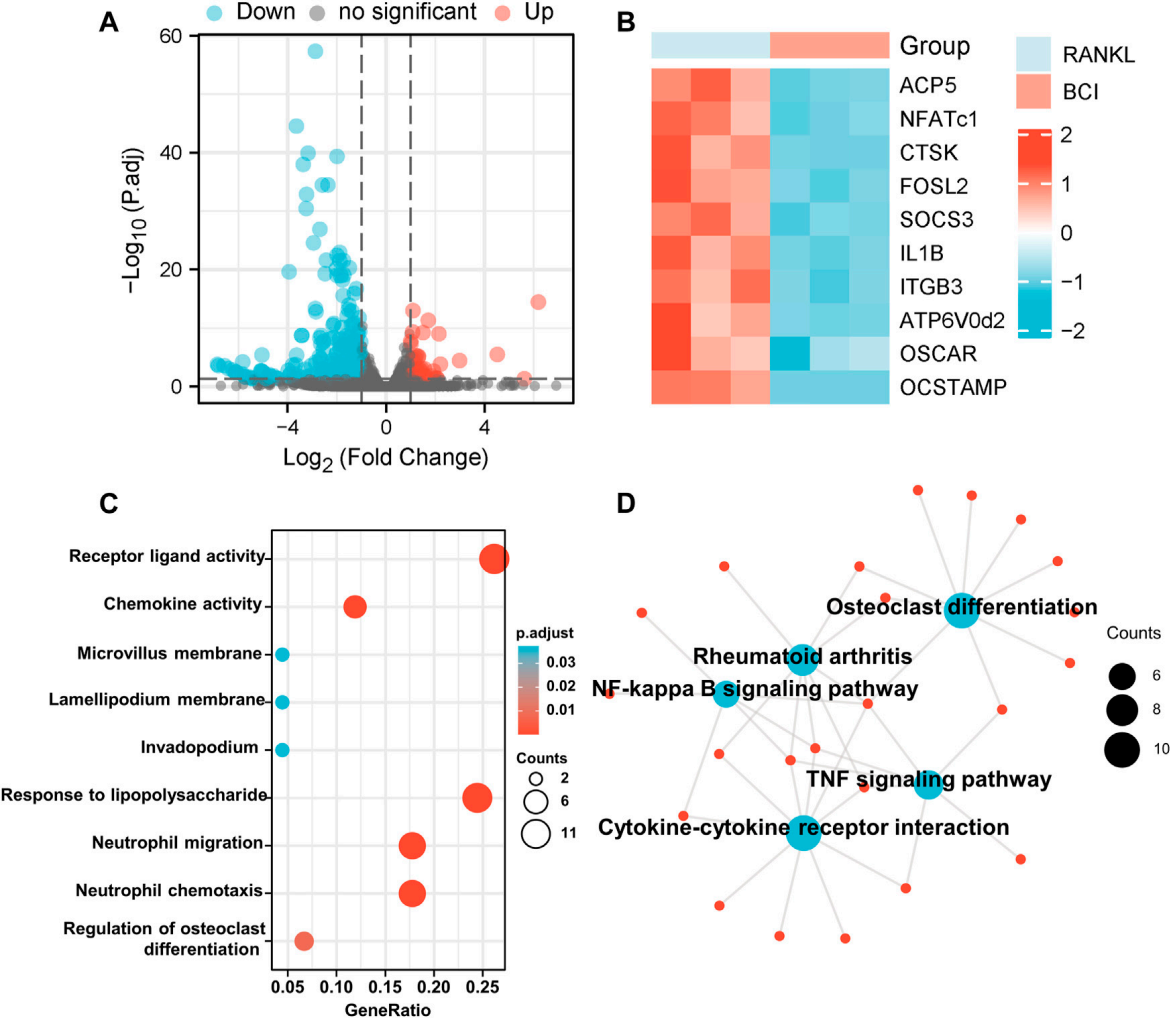
Transcriptome analysis of BCI-treated osteoclast differentiation. **(A)** The distribution of DEGs is presented as a volcano plot. **(B)** Heatmap illustrating differences in the expression of osteoclast-specific genes between RANKL-induced BCI-treated and -untreated groups via transcriptome analysis. **(C,D)** GO and KEGG enrichment results for the top 50 DEGs between RANKL-induced BCI-treated and -untreated groups. *p < 0.05, **p < 0.01, ***p < 0.001.

To elucidate the mechanism associated with BCI-mediated inhibition of osteoclastogenesis, the transcriptome of RANKL-induced RAW264.7 cells treated with or not with BCI was determined by RNA-seq analysis. The results identified 177 downregulated genes and 45 upregulated genes following BCI treatment. Figure 4A shows a volcano plot illustrating the distribution of DEGs. Moreover, RNA-seq analysis indicated that the expression of osteoclast-specific genes was significantly diminished following BCI treatment (Figure 4B), which was consistent with the qRT-PCR results.

We then performed GO and KEGG enrichment analysis to predict the functions and pathways associated with the 50 most significant DEGs under BCI treatment. The biological processes for these genes were predominantly enriched in receptor-ligand activity, chemokine activity, and regulation of osteoclast differentiation. The molecular functions for these genes mainly involved responses to LPS, neutrophil migration, and neutrophil chemotaxis (Figure 4C). The results of KEGG enrichment revealed the involvement of several major pathways, including those related to cytokine–cytokine receptor interaction, osteoclast differentiation, rheumatoid arthritis, and NF-κB and TNF signaling pathways (Figure 4D). These findings confirmed that BCI inhibited osteoclast differentiation and implicated several downstream signaling pathways.

### BCI Represses RANKL-Induced STAT3 and NF-ĸB–NFATc1 Signaling

**FIGURE 5:**
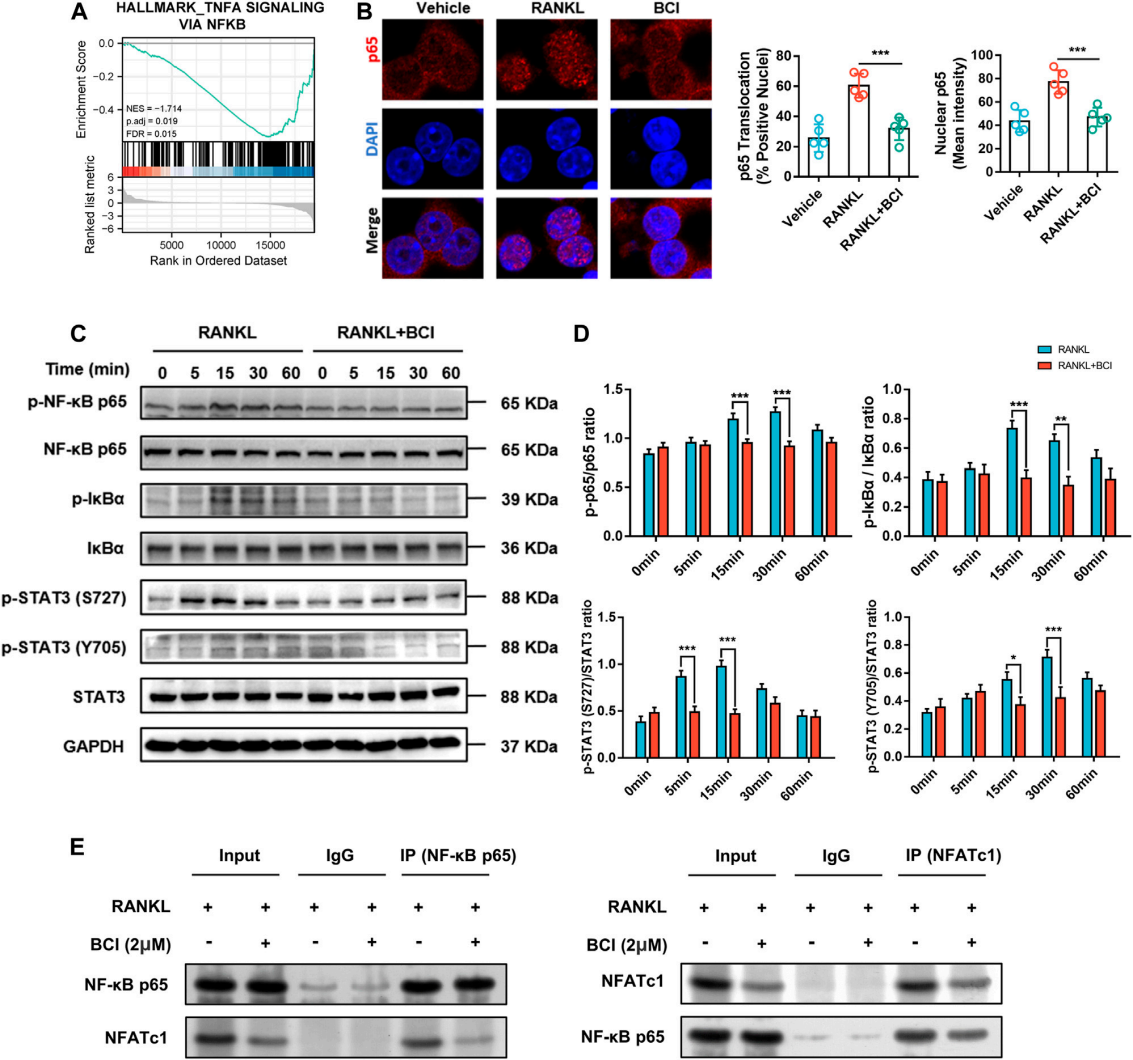
BCI suppresses RANKL-induced osteoclastogenesis via inhibiting NF-κB–NFATc1 interaction. **(A)** GSEA results showing altered TNF-α signaling via NF-κB expression and activation (adjusted *p* = 0.019; FDR *q*-value = 0.015). **(B)** Immunofluorescence staining of p65 during RANKL-induced osteoclastogenesis and with or without BCI treatment. **(C,D)** Western blot and corresponding quantification results showing levels of IκBα, p-IκBα, p65, p-p65, STAT3, and p-STAT3 at various time points (0, 5, 15, 30, and 60 min) in the control and BCI-treated groups. **(E)** Co-IP to assess NF-κB/p65 interaction with NFATc1. GAPDH was selected as the internal reference. *p < 0.05, **p < 0.01, ***p < 0.001.

Based on the results of KEGG pathway analysis (Figure 4D), we performed GSEA, with the results revealing significant enrichment of the TNF-α signaling via NF-κB in RANKL-induced cells but relatively little enrichment in cells cultured with BCI (Figure 5A). Additionally, immunofluorescence staining of NF-κB/p65 showed that nuclear translocation of NFκB/p65 was significantly upregulated following RANKL induction, whereas the presence of BCI suppressed this process (Figure 5B).

STAT3 can induce NF-κB activity and subsequently activate transcriptional regulation of downstream osteoclast-related genes, including CTSK and TRAP (Fan et al., 2013; Hu et al., 2020). Therefore, we detected STAT3 and NF-ĸB activities by western blot using induced RAW264.7 cells treated with or not with BCI (2 μM) for various times periods (5, 15, 30, and 60 min). Figure 5C shows that BCI significantly attenuated Ser727 and Tyr705 phosphorylation of STAT3 at 5, 15, and 30 min relative to that observed in the absence of BCI treatment. Additionally, phosphorylation of the p65 subunit and IκBα was suppressed by BCI at 15 and 30 min relative to that observed in the absence of BCI treatment (Figures 5C,D).

**FIGURE 6:**
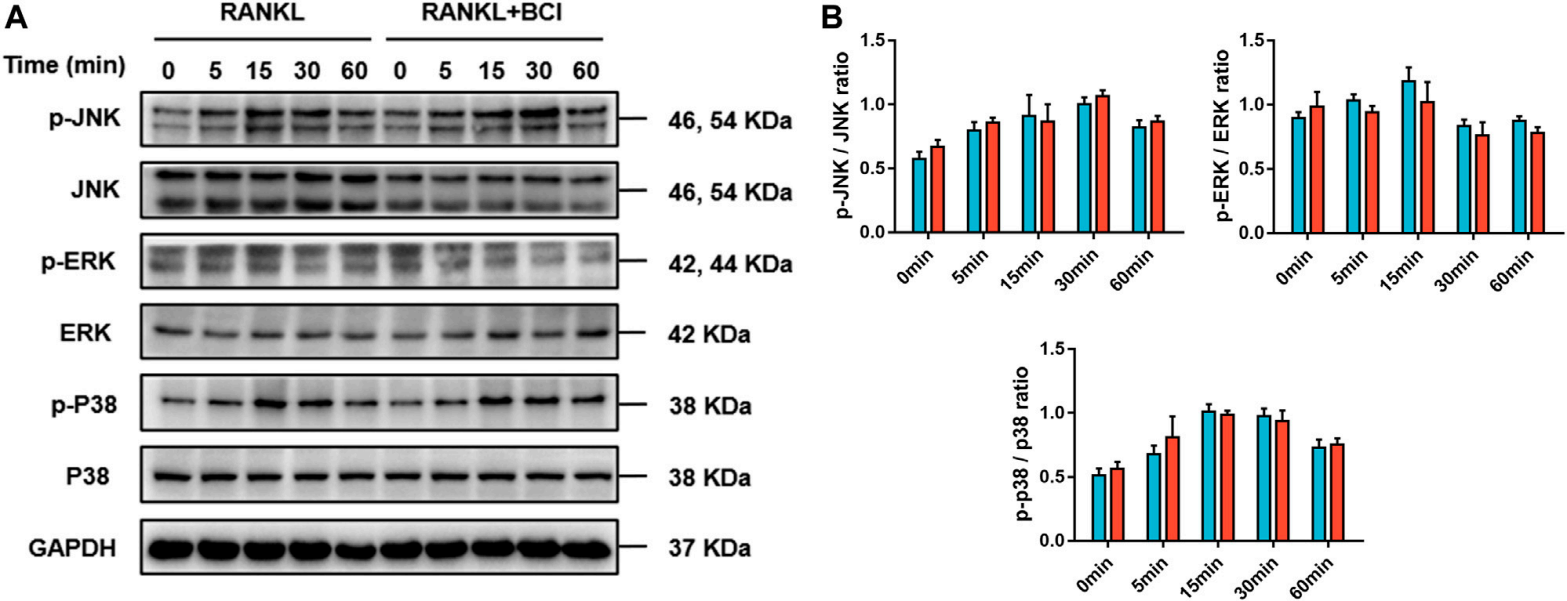
BCI suppresses RANKL-induced osteoclastogenesis independent of MAPK signaling. **(A,B)** Western blot and corresponding quantification results showing levels of JNK, p-JNK, ERK, p-ERK, p38, and p-p38 at various time points (0, 5, 15, 30, and 60 min) in the control and BCI-treated groups. GAPDH was selected as the internal reference. *p < 0.05, **p < 0.01, ***p < 0.001.

NFATc1 is a significant modulator of genes related to osteoclast differentiation (Liu et al., 2019). Our previous results in Figure 4 revealed that NFATc1 expression was downregulated following BCI treatment in a concentration- and time-dependent manner. Moreover, Co-IP to investigate interactions between NF-ĸB/p65 and NFATc1 indicated that this interaction was repressed under BCI treatment (Figure 5E). We then investigated whether inhibiting DUSP6 restrains MAPK signaling by measuring levels of phosphorylated p38, JNK, and ERK, with the results showing no changes in p-p38, p-JNK, and p-ERK levels under BCI treatment (Figures 6A,B). These results revealed that BCI inhibited RANKL-mediated osteoclast differentiation possibly by altering STAT3 and NF-ĸB–NFATc1 signaling.

### BCI Does Not Affect Osteoblast Differentiation or Mineralization

**FIGURE 7:**
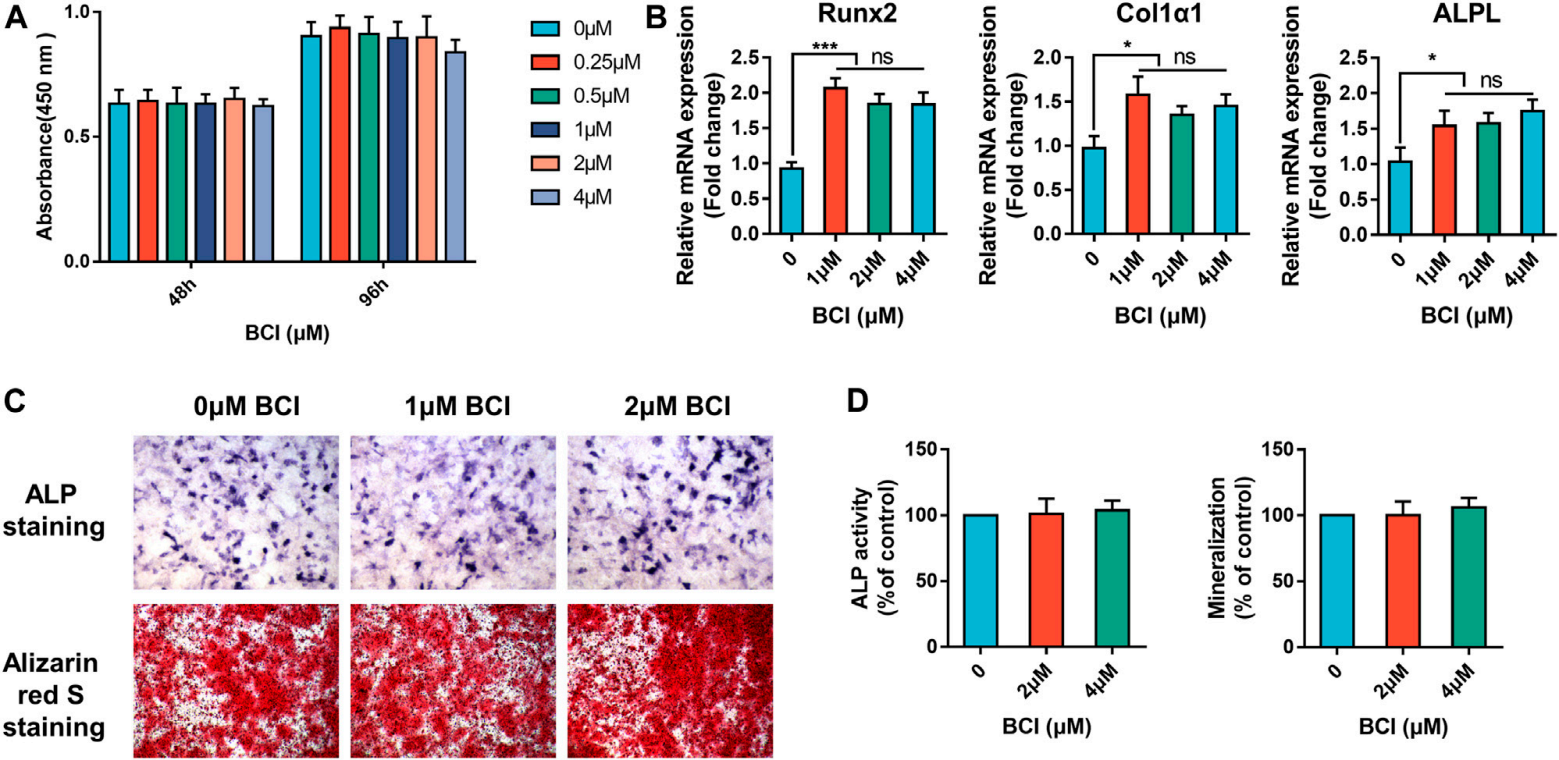
BCI does not affect osteogenic differentiation or mineralization. **(A)** CCK-8 evaluation of MC3T3-E1 cell viability following treatment with or without various doses of BCI for 48 and 96 h **(B)** qRT-PCR analysis of mRNA levels of key osteogenic differentiation genes (RUNX2, COL1α1, and ALPL) following BCI treatment. **(C,D)** Representative images of ALP and mineralization activity and corresponding quantification results in MC3T3-E1 cells induced by osteogenesis-inducing fluid and treated with or without different doses of BCI.

In addition to osteoclasts, bone-forming osteoblasts are critical regulators of bone homeostasis (Choi et al., 2013). First, we verified that BCI was not cytotoxic to the MC3T3-E1 cell line by CCK-8 assay, with the data indicating no significant changes in cell viability after 48 and 96 h of treatment with BCI (<4 µM) (Figure 7A). We then evaluated the effect of BCI on mRNA levels of key osteogenic differentiation genes (RUNX2, COL1α1, and ALPL). qRT-PCR results showed that BCI (<4 µM) treatment did not significantly alter the expression of these osteoblast-specific genes (Figure 7B). Moreover, we evaluated osteogenesis and mineralization of MC3T3-E1 cells under BCI treatment through ALP and alizarin red S staining. As expected, the results revealed no significant change between the control and BCI groups (Figures 7C,D). The above results demonstrated that BCI effectively inhibited osteoclast differentiation without affecting osteogenic differentiation.

### BCI Ameliorates OVX-Induced Bone Loss

**FIGURE 8:**
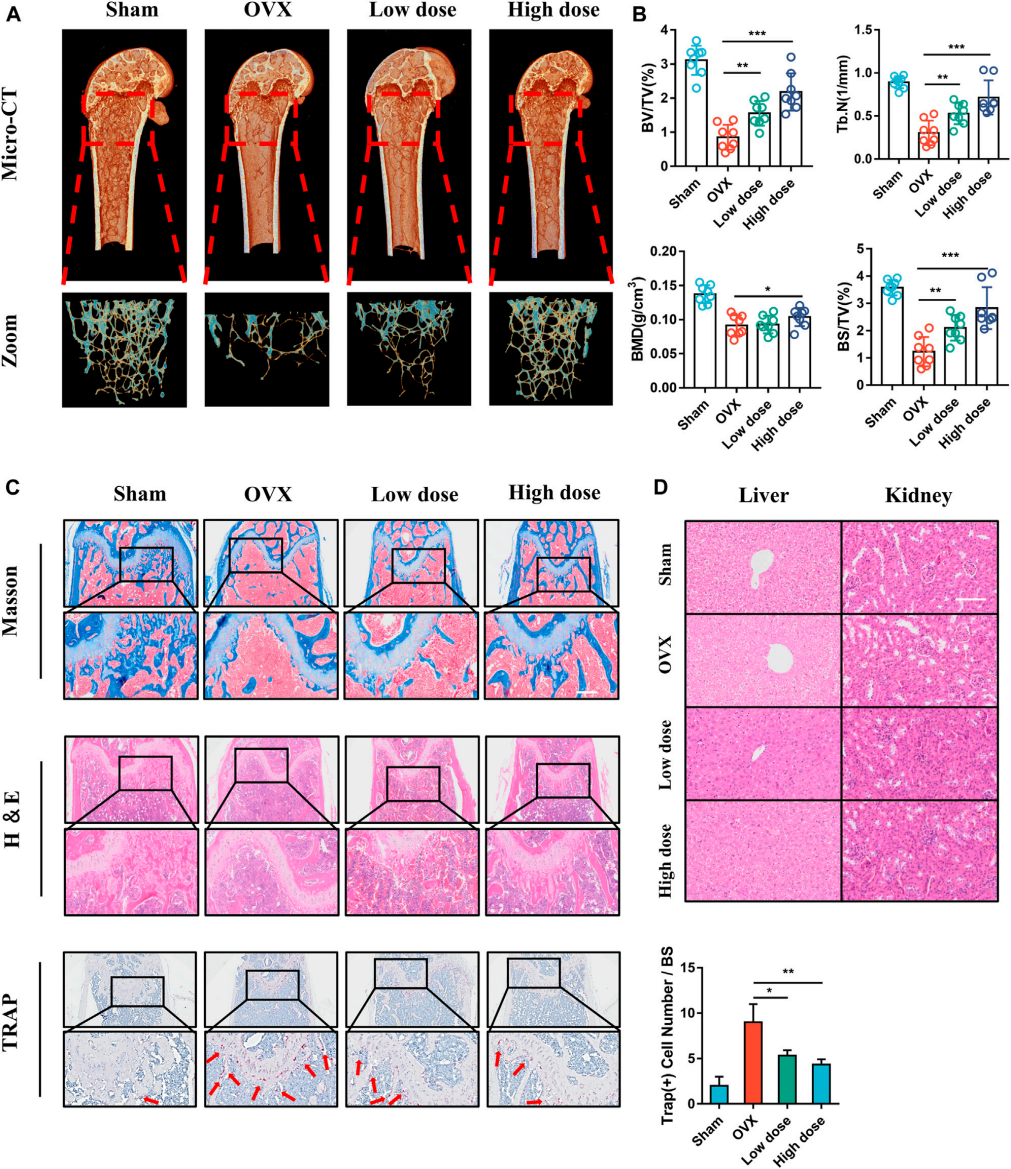
BCI protects against OVX-induced osteoporosis in mice. **(A)** Micro-CT images and 3D reconstruction of femurs from mice in the Sham, OVX, OVX + low-dose BCI, and OVX + high-dose BCI groups. **(B)** Statistical analysis of the bone parameters BV/TV, Tb.N, BMD, and BS/TV. **(C)** Representative images of histomorphologic analysis, including H&E, Masson, and TRAP staining. **(D)** H&E staining showing pathological changes in the liver and kidney of mice from each group.

**FIGURE 9:**
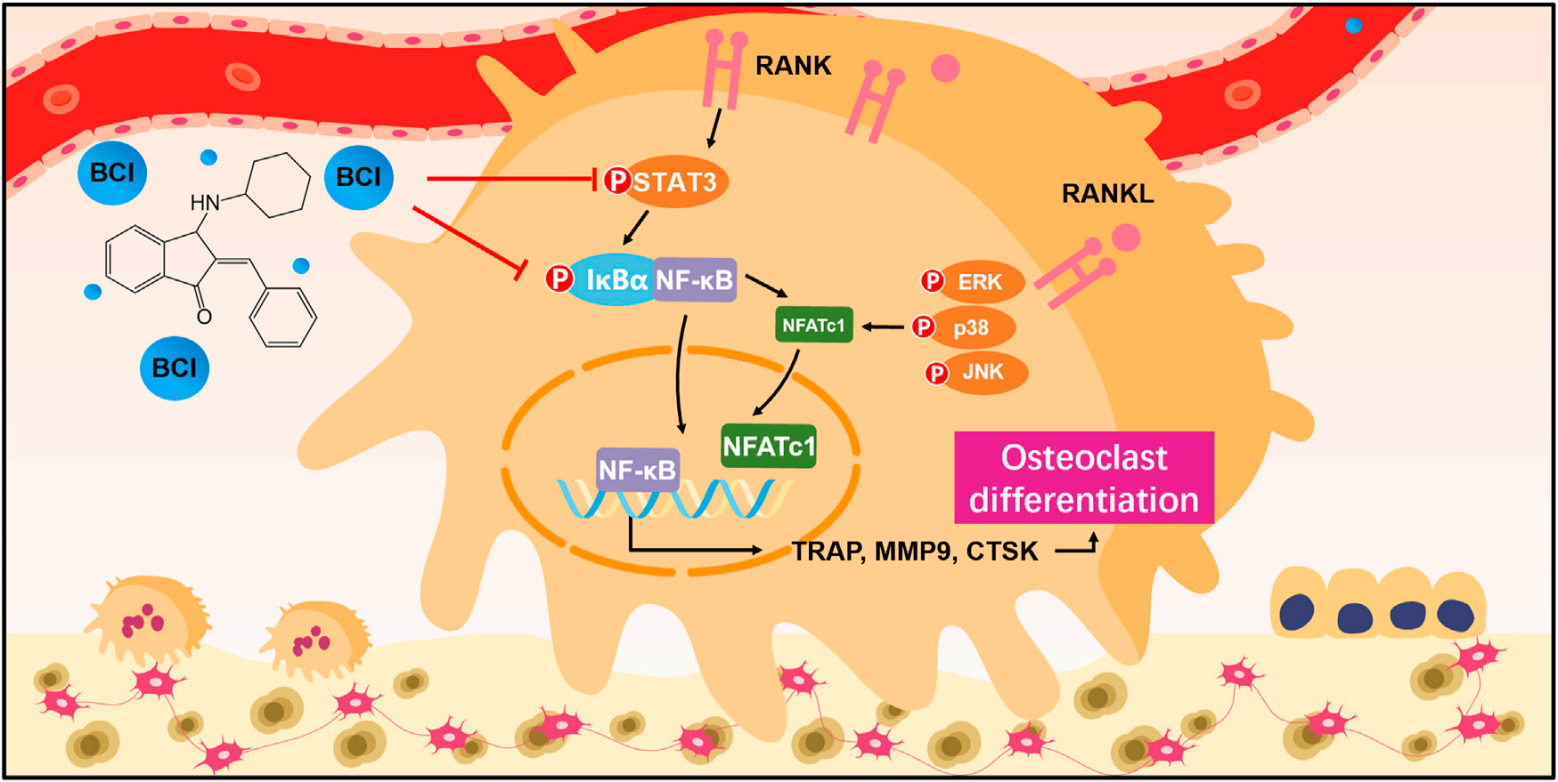
Schematic showing BCI-mediated regulation of RANKL-induced osteoclast differentiation and amelioration of OVX-induced osteoporosis.

We subsequently evaluated the effect of BCI *in vivo* following establishment of an OVX-induced osteoporotic mouse model. Low- or high-concentration (15 mg/kg or 30 mg/kg) BCI was injected intraperitoneally for 8 weeks, and bone loss was evaluated by micro-CT. We observed that bone loss was prevented in both the low- and high-concentration BCI groups (Figure 8A). Moreover, quantitative results indicated obvious increases in bone volume/total tissue volume (BV/TV), trabecular number (Tb.N), bone mineral density (BMD), and bone surface density (BS/TV) in both BCI-treated groups relative to the OVX group (Figure 8B). Consistent with the micro-CT results, H&E and Masson staining revealed obvious bone destruction in samples from the OVX group, whereas this was rarely observed in samples from the BCI-treated groups **(** Figure 8C). Furthermore, TRAP staining detected significant amounts of TRAP + cells in samples from the OVX group relative to Sham samples, and BCI treatment at both concentrations reduced the number of TRAP + osteoclasts per bone surface relative to the OVX group (Figure 8C). To assess hepatorenal toxicity under BCI treatment, we performed histologic analysis of the liver and kidney. The liver of mice from the sham and OVX group showed a normal structure, portal triad structure, and a central vein, whereas no apparent pathological changes were observed in the liver structures of BCI-treated mice (Figure 8D). Similarly, we observed no obvious pathological changes in kidney structures in BCI-treated animals (Figure 8D). These results suggested that BCI exhibited protective effects against OVX-induced osteoporosis by inhibiting osteoclast activation without significant hepatorenal toxicity. Schematic showing BCI-mediated regulation of RANKL-induced osteoclast differentiation and amelioration of OVX-induced osteoporosis was shown in Figure 9.

## Discussion

Osteoporosis is a common and frequent skeletal disorder in the elderly and mainly characterized by increased bone resorption, destruction of bone microstructure, and higher fracture risk (Sobacchi et al., 2013). Increases in age correlate with increases in the rate of osteoporosis, which seriously harms patient health (Nakashima et al., 2012). Bone remodeling and reconstruction are mainly achieved through osteoclast-regulated resorption and osteoblast-mediated bone formation (Henriksen et al., 2011). Maintaining a dynamic balance of bone-remodeling processes is essential for bone homeostasis. Osteoporosis occurs when bone resorption is greater than bone formation (Choi et al., 2013). Current first-line drugs for osteoporosis, such as bisphosphonates, mainly target osteoporosis caused by increased bone resorption (Leder et al., 2015); however, numerous studies report serious complications. For example, long-term use of chloromethyl bisphosphonate can lead to kidney damage, jaw lesions, esophageal cancer, and other serious consequences (Yuan et al., 2019). Thus, it is important to develop safe and effective anti-bone-resorption drugs to treat osteoporosis.

In the present research, we deeply explore the effect of BCI on osteoclastogenesis, revealing that BCI significantly inhibited osteoclast formation. Additionally, BCI inhibited osteoclast differentiation in a time and concentration-dependent manner. Bioinformatics analysis and experimental verification suggested that BCI inhibited osteoclastogenesis by altering STAT3 and NF-kB–NFATc1 signaling. Moreover, *in vivo* results showed that BCI treatment mitigated bone loss in OVX mouse model.

DUSP6 is a member of the MAPK phosphatase family and is responsible for dephosphorylating molecules at specific amino acid residues (Arkell et al., 2008). Recent research on mouse embryonic development showed that DUSP6 is a negative-feedback modulator of FGF receptor (FGFR)- mediated signaling, and that DUSP6 mutations are causal candidates for unexplained cases of FGFR-like syndromes. Regarding inflammation, DUSP6 reportedly promotes the inducible expression of intercellular adhesion molecule-1 *via* NF-κB activity in primary human endothelial cells, thereby promoting endothelial inflammation (Hsu SF. et al., 2018). Regulation of DUSP6 activity may be an effective clinical treatment modality.

BCI is a small-molecule inhibitor that reportedly attenuates DUSP6 levels and has been reported to have many biological functions such as tumor suppression and anti-inflammatory (Li et al., 2007; Hsu WC. et al., 2018; Wu et al., 2018). An *in vivo* study first reported the effects of BCI on zebrafish and revealed that BCI promotes the expression of fibroblast growth factor (FGF) in zebrafish embryos (Molina et al., 2009). Wu et al. (2018) reported that elevated DUSP6 expression is related to unfavorable prognosis in gastric tumors and that inhibiting DUSP6 using BCI markedly induced gastric cancer cell apoptosis and suppressed its proliferation. Additionally, an *in vitro* study found that BCI attenuates lipopolysaccharide (LPS)-induced proinflammatory responses in macrophages by activating Nrf2 (Zhang et al., 2019). Due to its close association with inflammatory signals, we hypothesized that BCI might affect osteoclast differentiation.

Our research demonstrated that BCI significantly inhibited osteoclast formation, fusion, and bone resorption according to TRAP staining, FAK staining, and pit-formation assays in BMMs and RAW 264.7 cells.

When RANKL binds to RANK located in preosteoclasts, osteoclastic differentiation is triggered by activation of downstream signals, including those involving c-Fos and NFATc1 (Luo et al., 2016). NFATc1 is a significant modulator of osteoclast-specific gene transcription, including TRAP, CTSK, and MMP9.

In our research, we found that BCI repressed c-Fos and NFATc1 expression in a concentration- and time-dependent way, as well as significantly downregulated the expression of the osteoclast marker genes such as CTSK and MMP9.

During osteoclastogenesis, RANKL–RANK interaction recruits TRAF6 (Walsh and Choi, 2014), followed by formation of the RANKL–RANK–TRAF6 complex, which activates TAK1 and initiates downstream MAPK and NF-κB signaling (Strickson et al., 2017). To explore the downstream molecular events of BCI-mediated inhibition of osteoclast differentiation, we used BCI to intervene in RANKL-induced osteoclastogenesis and performed transcriptome analysis. KEGG analysis of the identified DEGs indicated marked enrichment in pathways related to osteoclast differentiation and NF-κB activity, with GSEA also indicating significant downregulation of TNF-α signaling via NF-κB activation following BCI treatment.

NF-κB activation requires IκBα phosphorylation by IκB kinase, which promotes NF-κB/p65 translocation to the nucleus to promote transcriptional activity by factors, such as NFATc1 targeting osteoclast-specific genes (de la Rica et al., 2015). In the present research, we revealed that BCI treatment reversed RANKL-induced nuclear translocation of NF-κB/p65 via suppressed phosphorylation of IκBα. Moreover, co-IP assays demonstrated that BCI attenuated the interaction between NF-κB/p65 and NFATc1. In summary, we speculated that BCI inhibits NFATc1 expression by suppressing the activation and nuclear translocation of NF-κB/p65, thereby inhibiting osteoclastogenesis. Furthermore, MAPK signaling is critical for osteoclastogenesis (Suda et al., 1999); however, in our research, western blot results showed that BCI treatment inhibited osteoclast differentiation independent of MAPK signaling. Additionally, STAT3 participates in osteoclast formation and bone homeostasis by modulating chronic inflammation (Walton et al., 2002). A recent study reported that STAT3 binds the promoter region of NFATc1 to activate its transcription, and that deletion of STAT3 inhibited osteoclast maturation (Yang et al., 2019). In the present study, we revealed that BCI treatment reduced STAT3 activity in RANKL-mediated osteoclastogenesis, suggesting that BCI inhibited c-Fos and NFATc1 expression during osteoclastogenesis by restraining NF-kB and STAT3 activities.

We next assessed the *in vivo* effects of BCI on osteoporosis in a mouse model. Micro-CT data revealed that BCI treatment protected bone mass according to increases in BS/TV, BV/TV, Tb.N, and BMD in the trabecular bone area of OVX mice. Additionally, TRAP staining showed marked reductions in the number of TRAP + mature osteoclasts in trabeculated tissue of the distal femur following BCI treatment. Moreover, no obvious hepatotoxicity or nephrotoxicity was noted in BCI-treated mice. Furthermore, given that osteoblasts are critical regulators of bone homeostasis (Hu and Olsen, 2016), we evaluated their response to BCI treatment. ALP staining, alizarin red S staining, and qRT-PCR results demonstrated that BCI did not affect osteoblast differentiation.

Although we revealed the potential significance and possible mechanism of BCI in osteoclast formation and differentiation, there are still few questions that need to be explored and answered. First, a recent study found that DUSP6 expression is down-regulated in spinal osteoporotic tissues and (E/Z)-BCI promotes osteoclast formation via the ERK pathway (Zhang et al., 2021). Interestingly, however, in another study on macrophage inflammation, Zhang et al. found that DUSP6 was expression upregulated in LPS-activated RAW264.7 cells and (E/Z)-BCI inhibits LPS-induced macrophage inflammation in monocyte macrophages by inhibiting the NF-KB pathway (Zhang et al., 2019). Consistent with the results of Zhang et al., our results indicated that BCI hydrochloride significantly inhibited RANKL-induced osteoclasts differentiation via STAT3-NF-κB signaling. The reasons for this difference are not fully established. One likely explanation is that BCI is divided into different configurations (E configuration in our study). Drugs with different configurations may have different biological effects. Besides (E-Z)-BCI and BCI hydrochloride, although both DUSP6 inhibitors, may also inhibit other DUSP family members and may influence osteoclast formation. The effect of BCI on other DUSP family members will need to be investigated in subsequent studies.

This article also has the following limitations. Although we employed the BCI to evaluate its functions in osteoclastogenesis and the underlying mechanisms, the specific target and deeper mechanism of BCI inhibiting osteoclast differentiation remain to be further studied. Bone homeostasis is an active process involving bone resorption and formation; therefore, the precise effect of BCI *in vivo* osteogenic differentiation requires further investigation. Besides, due to technical difficulties and limited funding, our animal experiments only focused on the changes of the femur and ignored the influence of BCI on the vertebra. In terms of pharmaceutical evaluation, we lack the use of positive controls agents such as bisphosphonates to better evaluate the value of BCI in inhibiting osteoclast differentiation, which needs to be improved in the future; although we focused on the role of BCI in osteoclast differentiation and the corresponding potential molecular mechanism, we did not perform independent pharmacokinetic studies, which is also one of our limitations.

In summary, we revealed that BCI inhibited RANKL-mediated osteoclastogenesis by restraining STAT3 activity and NF-κB–NFATc1 signaling *in vitro* and ameliorating OVX-induced osteoporosis *in vivo*. These results indicated that targeting BCI treatment might represent a novel treatment strategy for osteoporosis.

## Data Availability

The data presented in the study are deposited in the NCBI SRA repository, accession number PRJNA769982.

## Abbreviations

ALP, alkaline phosphatase; BCI, BCI hydrochloride; BMD, bone mineral density; BMMs, bone marrow monocytes/macrophages; BS/TV, bone surface density; BV/TV, bone volume/total tissue volume; Co-IP, co-immunoprecipitation; DEG, differentially-expressed gene; DUSP6, dual-specificity phosphatase 6; FAK, focal adhesion kinase; FGF, fibroblast growth factor; GO, Gene Ontology; GSEA, Gene Set Enrichment Analysis; KEGG, Kyoto Encyclopedia of Genes and Genomes; MAPK, mitogen-activated protein kinase; M-CSF, macrophage colony-stimulating factor; NFATc1, nuclear factor of activated T cells 1; NF-κB, nuclear factor-kappaB; OVX, ovariectomy; RANKL, receptor activator of nuclear factor-kappaΒ ligand; STAT3, signal transducer and activator of transcription 3; Tb.N, trabecular number.
