# Supplementary material for: BCI Suppresses RANKL-Mediated Osteoclastogenesis and Alleviates Ovariectomy-Induced Bone Loss
Source: Front Pharmacol. 2021 Nov 1;12:772540. doi: 10.3389/fphar.2021.772540 (PMC8596812; doi:10.3389/fphar.2021.772540)
Supplement: Supplementary file 1 [file Table1.DOCX]

**Table S1.** Primer sequences for qRT-PCR.

| Genes | Forward | Reverse |
| --- | --- | --- |
| GAPDH | 5’-GGTTGTCTCCTGCGACTTCA-3’ | 5’-TGGTCCAGGGTTTCTTACTCC-3’ |
| DUSP6 | 5’-CGAGTCGTCACACATCGAATC-3’ | 5’-GCTATTCTCGTCGTACAGCAC-3’ |
| CTSK | 5’-GCTTGGCATCTTTCCAGTT-3’ | 5’-GTGCTTGCTTCCCTTCTG-3’ |
| c-Fos | 5’-GATGAGAAGTCTGCGTTGC-3’ | 5’-CTCTGGGAAGCCAAGGT-3’ |
| MMP9 | 5’-GACGACATAGACGGCATCC-3’ | 5’-TGGTTCAGTTGTGGTGGTG-3’ |
| NFATc1 | 5’-CCTTCAGAGAGACCTTGGC-3’ | 5’-CACAGGAGCTGGGGTTC-3’ |
| CD9 | 5’-AGTGGGTATCGGCATCG-3’ | 5’-GACCATTTCTCGGCTCCT-3’ |
| OSCAR | 5’-CGAAGGTTCTGGCTCCT-3’ | 5’-CCTGCTGTGCCAATCAC-3’ |
| PU.1 | 5’-TTACAGGCGTGCAAAATGGAA-3’ | 5’-GACGTTGGTATAGCTCTGAATCG-3’ |
| ATP6V0d2 | 5’-TGGCCTCATACGTTCATTT-3’ | 5’-TTTGAGCTTGGGGAGAAG-3’ |
| RUNX2 | 5’-GACTGTGGTTACCGTCATGGC-3’ | 5’-ACTTGGTTTTTCATAACAGCGGA-3’ |
| COL1α1 | 5’-GCTCCTCTTAGGGGCCACT-3’ | 5’-ATTGGGGACCCTTAGGCCAT-3’ |
| ALPL | 5’-CCAACTCTTTTGTGCCAGAGA-3’ | 5’-GGCTACATTGGTGTTGAGCTTTT -3’ |
